# Profiles of Risky Driving Behaviors in Adolescent Drivers: A Cluster Analysis of a Representative Sample from Tuscany Region (Italy)

**DOI:** 10.3390/ijerph18126362

**Published:** 2021-06-11

**Authors:** Vieri Lastrucci, Francesco Innocenti, Chiara Lorini, Alice Berti, Caterina Silvestri, Marco Lazzeretti, Fabio Voller, Guglielmo Bonaccorsi

**Affiliations:** 1 Epidemiology Unit, Meyer Children’s University Hospital, Viale Gaetano Pieraccini 24, 50139 Florence, Italy; 2 Department of Health Sciences, University of Florence, Viale GB Morgagni 48, 50134 Florence, Italy; chiara.lorini@unifi.it (C.L.); guglielmo.bonaccorsi@unifi.it (G.B.); 3 Epidemiologic Observatory, Regional Health Agency of Tuscany, Via Pietro Dazzi 1, 50141 Florence, Italy; francesco.innocenti@ars.toscana.it (F.I.); alice.berti@ars.toscana.it (A.B.); caterina.silvestri@ars.toscana.it (C.S.); marco.lazzeretti@ars.toscana.it (M.L.); fabio.voller@ars.toscana.it (F.V.)

**Keywords:** adolescent drivers, risky driving behaviors, cluster analysis, problem behaviors, road traffic accidents, severe road traffic accidents

## Abstract

(1) Background: Research on patterns of risky driving behaviors (RDBs) in adolescents is scarce. This study aims to identify distinctive patterns of RDBs and to explore their characteristics in a representative sample of adolescents. (2) Methods: this is a cross-sectional study of a representative sample of Tuscany Region students aged 14–19 years (*n* = 2162). The prevalence of 11 RDBs was assessed and a cluster analysis was conducted to identify patterns of RDBs. ANOVA, post hoc pairwise comparisons and multivariate logistic regression models were used to characterize cluster membership. (3) Results: four distinct clusters of drivers were identified based on patterns of RDBs; in particular, two clusters—the Reckless Drivers (11.2%) and the Careless Drivers (21.5%)—showed high-risk patterns of engagement in RDBs. These high-risk clusters exhibited the weakest social bonds, the highest psychological distress, the most frequent participation in health compromising and risky behaviors, and the highest risk of a road traffic accident. (4) Conclusion: findings suggest that it is possible to identify typical profiles of RDBs in adolescents and that risky driving profiles are positively interrelated with other risky behaviors. This clustering suggests the need to develop multicomponent prevention strategies rather than addressing specific RDBs in isolation.

## 1. Introduction

Road traffic accidents (RTAs) are a major public health issue worldwide and constitute the leading cause of death and acquired disabilities among young people of developed countries [1]. Among young people, adolescent drivers have the highest risk of experiencing an RTA because of their inexperience and the engagement in a wide variety of risky driving behaviors (RDBs) [2,3,4,5]. Indeed, adolescent and young drivers are particularly over-represented in RTAs involving RDBs such as distracted driving, speeding, fatigued driving and impaired driving states [6,7,8]. Compared with adult drivers, adolescents are more likely to engage in RDBs and tend to have a positive affect towards them [9].

While there is abundant research on antecedents, correlates and consequences of one RDB in isolation from other RDBs, there is relatively limited research on the concurrence of RDBs and its significance [10,11]. Evidence in this regard has highlighted that drivers that engage in a specific RDB often engage in other RDBs; for instances Tucker et al. found that speeding, texting while driving, and talking on the phone while driving co-occurs in adolescents, or Olsen et al. and Li et al. reported a positive correlation between the frequency of texting while driving and the prevalence of driving under the influence of drugs or of riding with a driver who had been drinking alcohol in adolescent drivers [12,13,14]. Nevertheless, research on how RDBs aggregate in adolescent populations and whether typical patterns of driving behaviors can be identified on that basis is scarce [15,16,17].

Cluster analysis (CA) is a promising approach to evaluate RDBs characteristics in a collective manner. CA enables subgroups (i.e., clusters) with shared characteristics to be identified within a population. In particular, this method categorizes a given population into mutually exclusive clusters with distinctive patterns of characteristics; individuals within a cluster have similar patterns of characteristics between them and are dissimilar to individuals belonging to other clusters [18,19]. While conducting a cluster analysis, the evaluation of precursors, correlates and risks of belonging to a specific cluster is a relevant part of the validation and characterization of profiles [20]. In the context of adolescent drivers and RDBs, such a holistic approach may help to identify and characterize RDB profiles and to better target traffic safety interventions to risk groups. Furthermore, it may contribute to the development of multicomponent public health interventions as risk-taking behaviors are frequently inter-related in adolescents and tend to share common precursors [15].

Thus far, limited research has explored clustering of RDBs to investigate patterns of RDBs in young drivers; this literature suggests that it is possible to distinguish different risk profiles of driving behavior in young drivers [16,17,21]. In particular, it appears to be a consensus that one or more profiles of problem driving behaviors exist among young drivers; however, there is no agreement regarding the pattern of RDBs and the specific characteristics—in terms of sociodemographic, mental health and well-being, and behaviors—associated with these profiles. Moreover, only few of the studies on this subject were carried out in a population-based representative sample of young people [21].

Therefore, the aim of this study is threefold. First, to evaluate the prevalence of numerous RDBs in a representative population-based sample of adolescent drivers of the Tuscany Region, Italy and to identify clusters of drivers that share similar patterns of RDBs. Second, if clusters of RDBs are present, to identify sociodemographic, mental and social well-being, and risky behaviors variables associated with cluster membership. Third, to evaluate the differences in the risk of RTAs across RDBs clusters. In the Tuscany Region, the categories of driving license follow the standard of the European Union; the minimum driving ages are: 14 years for two, three- or four-wheel mopeds with an engine capacity below 50 cc (license category AM); 16 years for motorbike of 50 cc to 125 cc (license category A2); and 18 years for passenger car (license category B) and motorbike over 125 cc (license category A2). Drivers are allowed to learn to drive a passenger car under supervision at age 17 years.

## 2. Materials and Methods

The study is based on data derived from the EDIT (Epidemiologia dei Determinanti dell’Infortunistica Stradale Toscana—Epidemiology of the determinants of traffic accidents in the Tuscany Region) surveillance system that was approved for research purposes by the Decree of the President of the Council of Ministers of Italy (Decreto del Presidente del Consiglio dei Ministri–DPCM) of 3 March 2017. The study was conducted according to the principles described in the Declaration of Helsinki.

### 2.1. Study Population

This is a cross sectional study based on the data provided by the 2018 EDIT surveillance survey (carried out from February to May). The EDIT surveillance survey investigates the epidemiology of RTAs and of their determinants in a representative sample of students attending the upper secondary schools of the Tuscany Region, Italy. The EDIT surveillance adopts a stratified sampling method according to the administrative areas of the Tuscany Region and of the type of secondary schools (i.e., lyceum, vocational college, technical and art college). An adequate sample size to estimate the population prevalence with a good precision was calculated using Epi Info software. Although, on the basis of the results of the previous EDIT survey (2015), the prevalence of variables of interest (e.g., RTAs, regular smokers, binge drinking and drug use) showed values sufficiently far from 50%, the sample size was calculated based on the most protective hypothesis of the maximum prevalence equal to 50%; assuming a maximum acceptable error of 5% for each stratum and a confidence level of 95%. The minimum sample size for each of the 26 sub-regional administrative areas (i.e., distretti sociosanitari) was established at 400 students. For each area of the region, schools were randomly selected and invited to participate in the surveillance system; for each of the included schools, at least one class per grade level was surveyed (upper secondary education in Italy consists of five grade levels, of which the first two are compulsory). A total of 85 upper secondary schools (21.9% of the upper secondary schools of the Region) were enrolled in the 2018 survey.

A total of 6824 students participated (response rate 96.6%) in the 2018 survey, representing the 3.55% of the population aged 14–19 in the Tuscany Region. For the purpose of the present study, only participants who reported driving at least once a week were considered (2764). In particular, drivers with a full driving license for the following type of vehicles were considered for the study: moped with an engine capacity below 50 cc; motorbike of 50 cc to 125 cc; motorbike over 125 cc, and passenger car.

### 2.2. Data Collection and Measurements

Students who gave their consent were asked to fill an anonymous self-administered questionnaire during school time. The survey questionnaire was administered via tablet devices allowing a real-time data collection.

The questionnaire consisted of 82 questions (average completion time of 45 min) and was divided into 11 sections covering the following topics: socio-demographic information, social well-being and mental health, driving behaviors and RTAs, health behaviors (smoking, alcohol consumption, recreational drugs consumptions, physical activity and dietary habits), and risk-taking behaviors (gambling, bullying, sexual behaviors, and risky riding behaviors). Each topic included in the questionnaire was developed using a tested methodology [22]. As far as the driving behaviors and RTA section is concerned, the type of motor vehicle used and the average frequency of driving (6–7 times a week, 2–5 times a week, once a week; coded 1–3) were evaluated. Furthermore, the following eleven RDBs were assessed: talking on phone while driving (TPWD); texting while driving (TWD); checking maps on the phone while driving; talking to passenger(s); smoking while driving; eating while driving; listening to loud music while driving; fatigued driving; speeding; driving under the influence (DUI) of alcohol; and DUI of recreational drugs. For each RDB, the frequency of participation over the last year was assessed, with the following response options provided for all the RDBs except for DUI of alcohol or drugs: never; a few times a month; several times a week; once a day; more than once a day (coded 1–5). DUI of alcohol and DUI of drugs were assessed with the following response options: never, once a month, a few times a month, a few times a week, several times a week (coded 1–5). Lastly, RTAs were measured as the number of RTAs—excluding minor crashes with very limited material damage—that occurred while driving a vehicle in life, and as the number of severe RTAs that occurred while driving a vehicle in life (defined as an RTA requiring the hospitalization of the driver).

Socio-demographic variables included sex, age, education level of the parents (pre-school education, primary education, lower secondary education, upper secondary education, bachelor’s degree, master’s degree or higher; coded 1–6), occupational status of parents and parental family status. As for social well-being indicators, the following variables were assessed: peer and family relationship (very poor, poor, fair, good, very good; coded 1–5), school performance (very poor, poor, fair, good, very good; coded 1–5) and school year failure (no, yes, coded 0–1).

Mental health-related variables included the occurrence during the last month of the following symptoms of psychological distress: nervousness, hopelessness, restlessness, sadness, exhaustion, and worthlessness (never, rarely, sometimes, often, very often, coded 1–5). Furthermore, sleep quality was assessed (deep/restful, light, interrupted; coded 1–3).

Health behaviors variables included smoking (regular smoker, occasional/social smoker, non-smoker; coded 1–3), being drunk in the last month (no, yes; coded 0–1), binge-drinking in the last month (defined as the consumption of five or more drinks in a row on an occasion; response options: no, yes; coded 0–1), use of at least one recreational drug in the last month (no, yes; coded 0–1), physical activity (defined as any bodily movement produced by skeletal muscles that require energy expenditure, response options: less than 1 time a week, 1 or more times a week; coded 0–1). As far as risk-taking behaviors are concerned, variables included bullying behavior in the last year (no, yes; coded 0–1), sexual intercourse initiation (no sexual initiation; after age 13; before age 13; coded 1–3), alcohol or recreational drug consumption before the last sexual intercourse (no, yes; coded 0–1), use of condom during the last sexual intercourse (no, yes; coded 0–1), and being a passenger of a driver under the influence of alcohol or recreational drugs in the last year (never, once a month, a few times a month, a few times a week, several times a week; coded 1–5). Lastly, gambling behavior was assessed with the Lie-Bet Screening Instrument [23] that allowed us to identify pathological gambling behavior according to the response to the following two questions: i. “Have you ever felt the need to bet more and more money?” ii. “Have you ever had to lie to people important to you about how much you gambled?” Participants with a positive response to one or both questions were considered at risk of pathological gambling behavior.

### 2.3. Statistical Analysis

Responses to the eleven RDBs evaluated in the survey were used to identify clusters with distinct profiles of risky driving. Subjects with missing values in one or more RDBs were not considered in the cluster analysis. The cluster analysis was performed with an approach involving two complementary steps. First, a hierarchical clustering with Ward’s method based on Euclidean distances was performed in 10 random samples of 10% of the study population. Ward’s analysis allowed for the identification of the possible appropriate solutions for the number of clusters; the possible solutions were determined through the visual examination of the dendograms produced and by the Calinski–Harabasz pseudo-F indices. Second, the appropriateness of the different solutions for the number of clusters was tested with a series of non-hierarchical cluster analyses (i.e., K-means) employed in the whole study population, and discriminant function analysis (DFA) was then employed to identify the optimal cluster solution. Composition of the clusters was described with K-means. As various different statistical approaches to cluster analysis exist and there are no defined guidelines, the above-described statistical approach was chosen in line with other studies carried out on the topic [16,17,21] so as to allow the comparability of the findings. The cluster solution was further validated by assessing the differences across clusters in (i) the frequency of engagement in each of the considered RDBs; (ii) the distribution of all the collected covariates; and (iii) the risk of RTAs and severe RTAs. In particular, one-way analyses of variance (ANOVA) were employed to evaluate whether significant differences in the distribution of RDBs and all the collected covariates existed across clusters; and Bonferroni test was used to perform post hoc pairwise comparison in order to identify significant differences in the participation of RDBs between specific clusters. As far as the differences in the risk of RTAs across clusters are concerned, two multivariable logistic regression models were performed. In the first model, the number of RTAs occurred while driving a vehicle in life—categorized as follows: no RTA in life vs. one or more RTAs in life—was used as dependent variables. In the second model, the risk of a severe RTA was analyzed using the number of RTAs requiring hospitalization as the dependent variable (i.e., no severe RTA in life vs. one or more severe RTAs in life). Both models were adjusted by age, sex, average driving frequency and type of motor vehicle used.

For each analysis, an α level of 0.05 was considered as significant. The statistical software STATA (Version 14.0; Stata Corporation, College Station, TX, USA) was used for data analyses.

## 3. Results

A total of 2764 participants reported driving at least once a week; of these, 2162 (78.2%) subjects provided information on all the RDB variables investigated and could therefore be included in the analyses. The sample characteristics are reported in Table 1. The mean age of the sample was 16.67 ± 1.35 years and males represented 68.1% of the sample. As for the average driving frequency, 64.1% of the sample reported driving six days a week/every day, whereas only 8.7% drove one day a week. A total of 714 participants (33.2%) reported to have had at least one RTA while driving, during the course of their life.

The prevalence of all the considered RDBs is reported in Table 1. As far as TPWD and TWD are concerned, 75.3% and 65% of the participants reported to have never engaged in these RDBs during the last year, respectively, whereas, 5.3% and 9.9% of participants reported a daily/more than once a day frequency of engagement in TPWD or TWD during the last year. DUI of alcohol or drugs during the last year occurred in the 12.5% and 11.2% of the sample, respectively; 0.7% and 2% of the sample reported a weekly or higher frequency of participation in DUI of alcohol or drugs, respectively. Talking to passengers, listening to loud music while driving, and speeding were found to be the RDBs with the highest weekly participation (Table 1).

Cluster analysis was used to determine the presence of patterns of driving behaviors among the participants. Inspection of the dendograms and the Calinski–Harabasz pseudo-F indices produced by the hierarchical cluster analyses suggested that a four-clusters solution was the most appropriate for the data set. K-means cluster analyses and DFA further confirmed the reliability of this cluster solution, with 98.1% of cases being correctly classified. The K-means of each of the considered RDBs across the four clusters is reported in Table 2.

Of the total sample, 45.56% was categorized as “Safe Drivers”, 21.79% as “Average Drivers”, 21.46% as “Careless Drivers”, and 11.19% as “Reckless and Impaired Drivers” (cluster names assigned by the authors). For each of the considered RDBs, the average frequency of engagement significantly differed across clusters (see Table 2); and the identified clusters were characterized by the following RDB patterns:

1. The Safe Drivers—this cluster is comprised of drivers who reported minimal or no engagement in all the considered RDBs; drivers in this cluster showed the lowest frequency of engagement in all the RDBs.
2. The Average Drivers—drivers in this cluster reported frequencies of engagement in speeding, fatigued driving, eating while driving, and checking maps on the phone while driving comparable to those of the sample population. Furthermore, drivers in this cluster generally refrained from TPWD, TWD, and DUI of alcohol or drugs. Lastly, a high rate of engagement in talking to other passenger(s) was observed in this cluster.
3. The Careless Drivers—this cluster is represented by drivers who presented moderate to high rates of engagement in all the distracted driving behaviors (i.e., TPWD, TXT, checking maps on the phone, smoking while driving, eating while driving, talking to passenger(s), and listening to loud music while driving) and speeding, but refrained from driving under the influence of drugs or alcohol.
4. The Reckless and Impaired Drivers (hereafter called reckless drivers)—this cluster is comprised of drivers who have the highest frequency of engagement in all the RDBs. In particular, drivers in this cluster reported several times a week to daily engagement in the following RDBs: TPWD, TWD, smoking while driving, listening to loud music, talking to other passenger(s) and speeding. Lastly, the participation in DUI of alcohol or drugs and fatigued driving in this cluster were significantly higher compared with all the other clusters, as shown by the post hoc pairwise comparisons between clusters.

Post hoc comparisons (Bonferroni test) confirmed that all pairwise comparisons for each of the RDBs were significantly different between clusters with few exceptions (6 out of 66 pairwise comparisons). In particular, no significant differences were found for the pairwise comparisons between Average Drivers and Careless Drivers in the following RDBs: smoking while driving, talking to passenger(s), fatigued driving, DUI of alcohol and DUI of drugs. Furthermore, the pairwise comparison between Safe Drivers and Average Drivers showed no significant differences in the frequency of engagement in listening to loud music while driving and DUI of alcohol or drugs.

The characteristics of the clusters in terms of socio-demographic, social well-being, mental health, health and risk-taking behaviors were examined; the variables that significantly differ across clusters are shown in Table 3, while in the Table S1 the characteristics of clusters for all the variables considered in the study are reported. As far as the demographic characteristics are concerned, the proportion of males was not significantly different among clusters, while age significantly differed among clusters, with the safe driver cluster presenting the lowest mean age. 

Considering the variables that significantly differed across clusters, it is possible to identify a pattern of the distribution of values across clusters; the safe drivers and the reckless drivers consistently presented the most extreme (highest/lowest) mean values in each of the variables considered, while the average drivers and the careless drivers presented intermediate values, respectively, closer to the values of the safe drivers and the reckless drivers clusters (see Table 3). In particular, the safe drivers cluster showed the lowest proportion of drivers with divorced parents (15.3%) and the most positive family relationships (mean value 4.39; proportion of drivers reporting good/very good family relationship: 90%) compared with other clusters (see Table 3 and Table S1). Furthermore, drivers in this cluster showed the highest school performances (mean value 3.76, proportion of drivers reporting good/very good school performance: 68.7%), least frequently experienced symptoms of psychological distress (see Table 3 and Table S1) and had the best sleep quality (mean value: 1.66; proportion of drivers reporting restful sleep 47.3%). Lastly, safe drivers showed the lowest frequencies of engagement in all the considered unhealthy lifestyle and other risk-taking behaviors (see Table 3 and Table S1), with the exceptions of impaired sex and bullying behaviors. In these two cases, safe drivers reported engaging in these behaviors less frequently (14% and 11%, respectively) than the average of the sample population (19% and 13%, respectively). Conversely, compared with other clusters reckless drivers had the highest proportion of drivers with divorced parents (22%), showed the poorest level of family relationships (mean value: 4.18; proportion of drivers reporting good/very good family relationship: 82%) and the lowest educational level of the father (mean value: 3.59; proportion of drivers with a father with bachelor’s degree or higher: 12.7%). Furthermore, reckless drivers were those who most frequently experienced symptoms of psychological distress in the last month—aside from the case of restlessness—and most frequently reported interrupted sleep (mean value of sleep quality: 1.82; proportion of drivers reporting interrupted sleep 26%). Finally, reckless drivers had the highest proportion of engagement in all the examined unhealthy lifestyle and risk-taking behaviors (see Table 3 and Table S1).

Table 4 reports the results of the logistic regression models for the risk of RTA and severe RTA. As for the risk of RTA, the careless and reckless drivers were about 1.8 and 3 times (OR 1.78; 95%CI 1.35–2.35 and OR 3.24; 95%CI 2.29–4.58, respectively) more likely to have experienced one or more RTAs while driving than safe drivers, respectively; no significant differences were observed in the odds of RTAs between the safe and average drivers. As for the risk of severe RTA, reckless drivers were about two times more likely to have experienced one or more severe RTAs while driving than safe drivers (OR 2.15, 95%CI 1.06–4.40). Compared with safe drivers, average and careless drivers showed no significant differences in the odds of severe RTAs. The extended results of the two logistic regression models are reported in Table S2.

## 4. Discussion

The aim of this study was to assess the prevalence of several RDBs and to evaluate whether it is possible to identify distinct patterns of RDBs in a representative sample of adolescent drivers of the Tuscany Region. Cluster analysis was used to identify patterns of RDBs among adolescent drivers, and the characteristics associated with cluster membership were studied: several socio-demographic, social well-being, mental health, health and risk-taking behaviors variables were analyzed. Lastly, the risk levels of an RTA and of a severe RTA were examined in order to identify differences across clusters. Results of the study showed that only a small proportion of adolescent drivers reported a high participation in most of the considered RDBs. Cluster analysis allowed the determination of the presence of four distinct profiles of driving behaviors, i.e., Safe Drivers, Average Drivers, Careless Drivers, and Reckless and Impaired Drivers. Drivers within each cluster had several common characteristics, and the distribution of these characteristics presented a consistent pattern across clusters. In particular, the highest and the lowest prevalence of all the considered symptoms of psychological distress, and of unhealthy and risk-taking behaviors were consistently observed in the reckless drivers and safe drivers, respectively. Lastly, results of the multivariable logistic regression models showed that the careless drivers and the reckless drivers had a higher risk of RTA while driving and that the reckless drivers also presented an increased risk of severe RTA.

Results of the cluster analysis highlighted the presence of four different patterns of driving behaviors in adolescent drivers; in particular, two very risky patterns of driving behaviors (i.e., the reckless and the careless drivers), one of moderate risk (the average drivers) and one safe (the safe drivers) pattern of driving behaviors were identified. As for the riskiest clusters, the reckless drivers showed the most deviant pattern with the highest degree of engagement in all the RDBs, while the careless drivers showed a pattern of co-occurrence of multiple RDBs, with a consistent participation in all the RDBs except for impaired driving behaviors. In contrast with these two risk-taking clusters, the safe drivers strongly refrained from all the RDBs. Notably, the results of the clustering of RDBs showed that the proportion of drivers who refrained from risky driving (the safe drivers) was similar to the proportion of adolescents who reported engagement—to varying degrees—in most RDBs (i.e., reckless, careless and average drivers). These findings indicate that adolescent drivers engage in multiple RDBs and not just in a single RDB and that there are two profiles of drivers that engage in them frequently. In this regard, our results confirm previous research reporting a positive correlation between specific RDBs in adolescents [12,13,14] and expand their conclusions suggesting the existence of broader and distinct patterns of co-occurrence of multiple RDBs among adolescent drivers. This clustering suggests the need to develop prevention strategies that simultaneously address different RDBs rather than focusing on one in particular.

As far as the distinctive characteristics associated with cluster membership are concerned, adolescents in the riskiest driving behavior patterns (i.e., the careless and the reckless drivers) exhibited weaker social bonds and more frequently experienced psychological distress; furthermore, they engaged in health-compromising behaviors and other risk-taking behaviors. These patterns of correlation provide evidence that these profiles of adolescent drivers may be part of a broader adolescent lifestyle which coherently fits within the problem behavior theory [24], which assumes that problem behaviors cluster in adolescents in a so-called “problem behavior syndrome” that is related to specific characteristics in the personality and perceived environment systems. Therefore, our findings confirm that problem driving behavior is an aspect and an indicator of a larger adolescent lifestyle characterized by several other problem behaviors such as problem drinking, drug use and bullying behavior [15,25,26].

Focusing on the pattern of correlations of the two risky driving profiles, careless drivers and reckless drivers seem to have distinct patterns of correlations, with weaker social bonds, greater substance use and more risky behavior proneness associated with the reckless drivers. These distinct patterns of correlations between the riskiest clusters well reflect the patterns of the RDBs characterizing them. Indeed, the reckless drivers showed a very high participation in all the RDBs, including substance-related driving behaviors that are a set of behaviors more socially and legally proscribed. The careless drivers, on the other hand, exhibited a pattern of RDBs that are more commonly observed among drivers and are less strongly prohibited by social values and legal norms. These observations suggest that careless drivers and reckless drivers are qualitatively distinct clusters in terms of compliance with social and legal norms. Other studies have observed that drinking-driving and drug-driving are predicted by weaker social bonds and more problem behavior proneness [15] compared with other risky driving behaviors. Our findings confirm and expand this understanding identifying two distinct high-risk patterns of adolescent drivers in terms of social prohibition, one more anti-social and one more normative. If confirmed by further studies, these patterns of characteristics associated with the cluster membership suggest the need to tailor preventative interventions according to the profiles of drivers, addressing “anti-social” risky drivers with multicomponent lifestyle interventions and “normative” risky drivers with interventions aimed at raising awareness on the risks involved in occasional participation in more conventional RDBs. Traditionally, the most widespread strategy to bring about change in conduct is to target risk behavior individually [27]. Nevertheless, our findings suggest that interventions that adopt a multi-risk approach—addressing several risk behaviors and unhealthy life habits simultaneously—may have a greater impact. Furthermore, integrated and coordinated preventive programs that intervene in multiple community settings (such as schools, parks, health clinics, and sport facilities), that involve the key stakeholders and adopt multiple components (such as, education, skills training, social marketing, and advocacy) at the same time are likely to be more effective and efficient in targeting RDBs than individual interventions [28,29,30].

Lastly, as far as the risk of RTA is concerned, results are congruent with the cluster solution and the characteristics associated with cluster membership. Furthermore, findings in the risk of RTA further confirm the qualitative distinction between the two risky driving profiles; the careless drivers cluster—with a moderate risk of RTA and no increases in the risk of severe RTA—appears to be a profile of intermediate severity between lower risk profiles (i.e., safe and average drivers) and the reckless drivers, which showed relevant risks of RTA and severe RTA.

The present study has several strengths and limitations. As for the strengths, this is one of the first studies assessing the prevalence of a comprehensive range of RDBs in a large and representative sample of adolescent drivers to date, and the study had a participation rate of 96.6%. Furthermore, the study has performed a novel and promising approach (i.e., cluster analysis) in the evaluation of adolescent driving behaviors that allowed the consideration of multiple RDBs in a collective manner and the identification of different profiles of RDBs in adolescent drivers. Furthermore, a broad range of driver characteristics of different content areas were considered. As far as the limitations are concerned, the study was based on self-reported data; therefore, it may have suffered from a recall and social desirability bias of participants. However, previous studies have shown that the extent of these biases is minimal in the context of self-reporting driving behaviors [31,32]; moreover, the self-administration and the anonymity of the survey may have further limited the potential social desirability bias. Lastly, the cross-sectional design of the study prevents the establishment of temporal relationships between RDB profiles and adolescent characteristics. Therefore, further longitudinal studies are needed to explore precursors and temporal sequence of appearance of the highlighted RDB profiles.

## 5. Conclusions

In conclusion, this is one of the first studies analyzing a comprehensive set of RDBs in a collective manner in a large and representative sample of adolescent drivers. Results showed that a relevant proportion of adolescents engage—to varying degrees—in several RDBs and that it is possible to identify four distinct profiles of RDBs in adolescent drivers. Of these profiles, two exhibited a high-risk pattern of engagement in RDBs, one reported an occasional engagement in multiple RDBs and one strongly refrained from the participation in all the RDBs. As far as the two identified profiles of high-risk drivers are concerned, one was characterized by a very high participation in all the RDBs considered including the substance-related driving behaviors, while the other one exhibited a more moderate participation in most of the RDBs and a minimal engagement in substance related-driving behaviors. Characteristics associated with cluster membership suggested that the two profiles of high-risk drivers were qualitatively distinct; the riskiest profile of drivers seems to be part of a more general behavioral lifestyle of normative transgression and risky behavior proneness, and the other one reflects a more normative and conventional tendency. Results of the study may allow us to better identify and target adolescents at higher risk of road traffic accidents. Furthermore, our findings have major implications for public health policies and interventions addressing adolescent risky driving behaviors; as risky driving behaviors seem to occur in specific patterns and are positively interrelated with other risky behaviors, they suggest the need to develop multicomponent prevention strategies rather than addressing a specific risky driving behavior in isolation.

## Figures and Tables

**Table 1:** Demographic characteristics and driving behaviors of the study population (*n* = 2.162).

	*n* (%)	Proportion of Male (%)	Mean Age (SD)
Sex			
*Male*	1473 (68.1)	100	16.67 (1.36)
*Female*	689 (31.9)	-	16.82 (1.33)
Average driving frequency *			
*6 day a week/every day*	1386 (64.1)	72.6	16.82 (1.33)
*2–5 days a week*	587 (27.2)	60.3	16.56 (1.37)
*1 day a week*	189 (8.7)	59.8	16.47 (1.41)
Talking on phone while driving °°			
*Never*	1628 (75.3)	67.1	16.53 (1.38)
*Few times a month*	288 (13.3)	70.8	17.17 (1.14)
*Several times a week*	131 (6.1)	69.5	17.31 (1.11)
*Once a day*	50 (2.3)	78.0	17.50 (0.95)
*More than once a day*	65 (3.0)	72.3	17.55 (0.90)
Texting while driving °°			
*Never*	1406 (65.0)	67.3	16.44 (1.39)
*Few times a month*	372 (17.2)	68.8	17.07 (1.16)
*Several times a week*	170 (7.9)	66.5	17.26 (1.15)
*Once a day*	113 (5.2)	75.2	17.47 (0.99)
*More than once a day*	101 (4.7)	72.3	17.46 (1.01)
Checking maps on the phone while driving °°			
*Never*	1724 (79.7)	68.8	16.55 (1.36)
*Few times a month*	345 (16.0)	64.9	17.34 (1.15)
*Several times a week*	67 (3.1)	62.7	17.52 (0.86)
*Once a day*	11 (0.5)	90.9	17.91 (0.30)
*More than once a day*	15 (0.7)	73.3	17.27 (1.03)
Smoking while driving °°			
*Never*	1798 (83.2)	67.7	16.61 (1.38)
*Few times a month*	133 (6.2)	66.2	16.95 (1.14)
*Several times a week*	75 (3.5)	66,7	17.27 (1.18)
*Once a day*	58 (2.7)	69.0	17.47 (0.78)
*More than once a day*	98 (4.5)	78.6	17.47 (0.93)
Eating while driving			
*Never*	1724 (79.7)	67.8	16.62 (1.36)
*Few times a month*	259 (12.0)	69.5	17.15 (1.18)
*Several times a week*	110 (5.1)	66.4	17.03 (1.38)
*Once a day*	34 (1.6)	61.8	17.29 (1.27)
*More than once a day*	35 (1.6)	85.7	16.86 (1.40)
Talking to passenger(s) °°			
*Never*	661 (30.6)	69.0	15.87 (1.31)
*Few times a month*	387 (17.9)	72,1	16.56 (1.23)
*Several times a week*	350 (16.2)	68.3	17.04 (1.20)
*Once a day*	279 (12.9)	65.6	17.27 (1.16)
*More than once a day*	485 (22.4)	65.2	17.45 (1.01)
Listening to loud music while driving °°			
*Never*	1188 (54.9)	70.3	16.36 (1.37)
*Few times a month*	234 (10.8)	66.7	16.86 (1.30)
*Several times a week*	238 (11.0)	66.4	17.04 (1.27)
*Once a day*	181 (8.4)	65.2	17.35 (1.08)
*More than once a day*	321 (14.8)	64.2	17.32 (1.09)
Fatigued driving °°			
*Never*	1291 (59.7)	67.5	16.51 (1.41)
*Few times a month*	731 (33.8)	68.1	17.02 (1.20)
*Several times a week*	111 (5.1)	70.3	16.95 (1.23)
*Once a day*	11 (0.5)	90.9	17.45 (1.04)
*More than once a day*	18 (0.8)	88.9	17.17 (1.04)
Speeding °°			
*Never*	741 (34.3)	67.,6	16.60 (1.48)
*Few times a month*	770 (35.6)	70.4	16.64 (1.32)
*Several times a week*	449 (20.8)	68.6	16.90 (1.23)
*Once a day*	108 (5.0)	61.1	16.99 (1.20)
*More than once a day*	94 (4.3)	59.6	17.04 (1.14)
Driving under the influence of alcohol * °°			
*Never*	1892 (87.5)	66.7	16.66 (1.38)
*Once a month*	213 (9.9)	75.6	17.08 (1.06)
*A few times a month*	42 (1.9)	85.7	17.19 (1.09)
*A few times a week*	3 (0.1)	66.7	17.33 (1.15)
*Several times a week*	12 (0.6)	100.0	17.25 (1.29)
Driving under the influence of drugs * °°			
*Never*	1919 (88.8)	67.0	16.68 (1.38)
*Once a month*	145 (6.7)	73.1	16.89 (1.14)
*A few times a month*	54 (2.5)	79.6	17.19 (0.93)
*A few times a week*	14 (0.6)	85.7	16.93 (1.44)
*Several times a week*	30 (1.4)	90.0	17.37 (1.03)
RTAs in life * °°			
*No*	1434 (66.8)	64.2	16.62 (1.43)
*One or more*	714 (33.2)	76.1	16.93 (1.16)

* Chi2 test for sex, *p* < 0.001 ° ANOVA for age, *p* < 0.05; °° ANOVA for age, *p* < 0.001.

**Table 2:** Cluster analysis (k-means): description of the 4-clusters solution and cluster comparisons (ANOVA and Bonferroni test).

Risky Driving Behaviors	Total	Safe Drivers	Average Drivers	Careless Drivers	Reckless and Impaired Drivers	F Score
*n* (%)	2162(100)	985(45.56)	471(21.79)	464(21.46)	242(11.19)	
Talking on phone while driving °						
Mean (SD)	1.44(0.93)	1.05(0.27)	1.28(0.59)	1.44(0.67)	3.36(1.26)	924.1 *
95%C.I.	1.40–1.48	1.04–1.07	1.23–1.33	1.38–1.50	3.21–3.52	
Texting while driving °						
Mean (SD)	1.67(1.12)	1.11(0.38)	1.49(0.77)	1.82(0.91)	4.03(0.95)	1185.1 *
95%C.I.	1.63–1.72	1.09–1.14	1.42–1.56	1.74–1.90	3.91–4.15	
Checking maps on the phone while driving °						
Mean (SD)	1.26(0.61)	1.06(0.27)	1.29(0.56)	1.41(0.67)	1.76(1.06)	114.6 *
95%C.I.	1.24–1.29	1.04–1.08	1.24–1.34	1.35–1.47	1.62–1.89	
Smoking while driving ^a^						
Mean (SD)	1.39(1.01)	1.07(0.36)	1.29(0.80)	1.42(0.95)	2.85(1.73)	285.8 *
95%C.I.	1.35–1.44	1.05–1.09	1.22–1.36	1.33–1.51	2.63–3.07	
Eating while driving °						
Mean (SD)	1.33(0.78)	1.09(0.38)	1.25(0.60)	1.39(0.71)	2.38(1.36)	240.7 *
95%C.I.	1.30–1.37	1.06–1.11	1.19–1.30	1.33–1.46	2.21–2.56	
Talking to passenger(s) ^a^						
Mean (SD)	2.79(1.54)	1.35(0.48)	3.86(0.87)	3.85(1.09)	4.52(0.88)	2090.8 *
95%C.I.	2.72–2.85	1.32–1.38	3.78–3.93	3.75–3.95	4.41–4.64	
Listening to loud music while driving ^b^						
Mean (SD)	2.17(1.52)	1.22(0.56)	1.24(0.43)	4.02(0.85)	4.31(1.05)	2904.2 *
95%C.I.	2.11–2.24	1.19–1.26	1.20–1.28	3.95–4.10	4.17–4.44	
Fatigued driving ^a^						
Mean (SD)	1.49(0.70)	1.27(0.51)	1.50(0.66)	1.58(0.62)	2.17(1.01)	133.0 *
95%C.I.	1.46–1.52	1.24–1.31	1.44–1.56	1.53–1.64	2.05–2.30	
Speeding °						
Mean (SD)	2.10(1.06)	1.76(0.81)	2.05(1.00)	2.28(1.10)	3.17(1.24)	141.5 *
95%C.I.	2.05–2.14	1.71–1.82	1.96–2.14	2.18–2.38	3.01–3.32	
Driving under the influence of alcohol ^c^						
Mean (SD)	1.16(0.50)	1.07(0.29)	1.13(0.38)	1.15(0.43)	1.64(1.00)	97.4 *
95%C.I.	1.14–1.18	1.05–1.09	1.10–1.16	1.11–1.19	1.51–1.77	
Driving under the influence of drugs ^c^						
Mean (SD)	1.19(0.64)	1.08(0.39)	1.12(0.44)	1.22(0.64)	1.74(1.23)	77.9 *
95%C.I.	1.16–1.22	1.06–1.10	1.08–1.16	1.16–1.28	1.58–1.89	

* *p* < 0.001; ANOVA test *df* (3, 2.162); ° Significant differences (*p* < 0.001) between the 4 clusters in all the pairwise post hoc comparisons (Bonferroni test); ^a^ Significant differences (*p* < 0.001) between the 4 clusters in all the pairwise post hoc comparisons with the exception of the pairwise comparison between Average Drivers and Careless Drivers; ^b^ Significant differences (*p* < 0.001) between the 4 clusters in all the pairwise post hoc comparisons with the exception of the pairwise comparison between Safe Drivers and Average Drivers; ^c^ Significant differences (*p* < 0.001) between the 4 clusters in all the pairwise post hoc comparisons with the exception of the pairwise comparisons between Safe Drivers and Average Drivers and between Average Drivers and Careless Drivers.

**Table 3:** Significant variables associated with the cluster solution (ANOVA).

Characteristics		Total	Safe Drivers	Average Drivers	Careless Drivers	Reckless and Impaired Drivers	*F* Score
Age (14–18 years)	Mean(SD)	16.72(1.35)	16.09(1.32)	17.1(1.2)	17.29(1.12)	17.44(0.98)	165.98 ***
	95%CI	16.66–16.77	16–16.17	16.99–17.21	17.19–17.39	17.32–17.57	
Education level of the father (1–6)	Mean(SD)	3.83(1.08)	3.91(1.11)	3.79(1.03)	3.85(1.06)	3.59(1.07)	5.63 ***
	95%CI	3.79–3.88	3.84–3.98	3.7–3.89	3.75–3.95	3.45–3.73	
Divorced parents (0–1)	Mean(SD)	0.18(0.38)	0.15(0.36)	0.19(0.39)	0.2(0.4)	0.22(0.42)	2.82 *
	95%CI	0.16–0.19	0.13–0.18	0.15–0.22	0.16–0.23	0.17–0.27	
Quality of family relationships (1–5)	Mean(SD)	4.34(0.83)	4.39(0.78)	4.34(0.84)	4.33(0.82)	4.18(0.99)	4.07 **
	95%CI	4.31–4.38	4.34–4.44	4.27–4.42	4.25–4.4	4.05–4.31	
School performances (1–5)	Mean(SD)	3.69(0.82)	3.76(0.79)	3.75(0.8)	3.67(0.82)	3.4(0.89)	12.58 ***
	95%CI	3.65–3.72	3.71–3.81	3.68–3.82	3.59–3.74	3.28–3.51	
School year failure (0–1)	Mean(SD)	0.23(0.42)	0.15(0.35)	0.22(0.42)	0.27(0.45)	0.47(0.5)	43.74 ***
	95%CI	0.21–0.24	0.12–0.17	0.18–0.26	0.23–0.31	0.41–0.53	
Nervousness in the last month (1–5)	Mean(SD)	3.14(0.96)	3.01(0.96)	3.17(0.93)	3.31(0.9)	3.3(1.02)	14.35 ***
	95%CI	3.1–3.18	2.95–3.06	3.09–3.26	3.23–3.4	3.17–3.43	
Hopelessness in the last month (1–5)	Mean(SD)	1.95(1.14)	1.87(1.11)	1.93(1.12)	2.04(1.13)	2.15(1.24)	5.28 **
	95%CI	1.9–2	1.8–1.94	1.83–2.03	1.94–2.14	1.99–2.31	
Restlessness in the last month (1–5)	Mean(SD)	2.73(1.07)	2.58(1.08)	2.77(1.01)	2.92(1.05)	2.85(1.11)	12.19 ***
	95%CI	2.68–2.77	2.52–2.65	2.68–2.86	2.82–3.01	2.71–2.99	
Exhaustion in the last month (1–5)	Mean(SD)	2.2(1.14)	2.12(1.12)	2.16(1.1)	2.32(1.14)	2.43(1.25)	6.71 ***
	95%CI	2.16–2.25	2.05–2.19	2.06–2.26	2.21–2.42	2.27–2.59	
Sleep quality (1–3)	Mean(SD)	1.72(0.73)	1.66(0.7)	1.73(0.72)	1.79(0.73)	1.82(0.82)	5.32 **
	95%CI	1.69–1.75	1.61–1.7	1.66–1.79	1.72–1.86	1.72–1.93	
Pathological gambling (0–1)	Mean(SD)	0.11(0.31)	0.08(0.27)	0.08(0.27)	0.12(0.33)	0.24(0.43)	20.22 ***
	95%CI	0.09–0.12	0.06–0.1	0.05–0.1	0.09–0.15	0.19–0.3	
Smoking habit (1–3)	Mean(SD)	2.36(0.85)	2.58(0.74)	2.42(0.82)	2.18(0.89)	1.71(0.87)	83.60 ***
	95%CI	2.32–2.4	2.53–2.62	2.34–2.49	2.1–2.26	1.6–1.82	
Being drunk in the last year (0–1)	Mean(SD)	0.59(0.49)	0.45(0.5)	0.62(0.49)	0.7(0.46)	0.85(0.36)	57.43 ***
	95%CI	0.57–0.61	0.42–0.49	0.57–0.66	0.65–0.74	0.8–0.9	
Binge drinking in the last month (0–1)	Mean(SD)	0.42(0.49)	0.31(0.46)	0.41(0.49)	0.5(0.5)	0.72(0.45)	50.77 ***
	95%CI	0.4–0.44	0.28–0.34	0.37–0.46	0.45–0.54	0.67–0.78	
Drug use in the last month (0–1)	Mean(SD)	0.24(0.43)	0.16(0.37)	0.25(0.43)	0.29(0.45)	0.49(0.5)	42.09 ***
	95%CI	0.23–0.26	0.14–0.18	0.21–0.29	0.25–0.33	0.43–0.56	
Bulling behavior in the last year (0–1)	Mean(SD)	0.13(0.33)	0.11(0.31)	0.09(0.29)	0.14(0.34)	0.24(0.43)	12.20 ***
	95%CI	0.11–0.14	0.09–0.13	0.07–0.12	0.11–0.17	0.18–0.29	
Age of first sexual intercourse (1–3)	Mean(SD)	1.64(0.69)	1.44(0.65)	1.67(0.66)	1.77(0.66)	2.16(0.65)	87.44 ***
	95%CI	1.61–1.67	1.4–1.48	1.61–1.73	1.71–1.83	2.08–2.24	
Use of alcohol or drug before the last sexual intercourse (0–1)	Mean(SD)	0.19(0.39)	0.14(0.35)	0.13(0.34)	0.17(0.38)	0.37(0.48)	18.25 ***
	95%CI	0.16–0.21	0.11–0.18	0.09–0.17	0.13–0.21	0.3–0.43	
Use of condom during the last sexualintercourse (0–1)	Mean(SD)	0.61(0.49)	0.7(0.46)	0.68(0.47)	0.55(0.5)	0.46(0.5)	13.96 ***
	95%CI	0.58–0.64	0.65–0.75	0.62–0.74	0.5–0.61	0.39–0.53	
Average frequency of driving (1–3)	Mean(SD)	1.45(0.65)	1.63(0.71)	1.37(0.59)	1.31(0.56)	1.12(0.35)	62.40 ***
	95%CI	1.42–1.47	1.59–1.68	1.31–1.42	1.25–1.36	1.07–1.16	
Passenger of a drunk driver (1–5)	Mean(SD)	1.24(0.54)	1.14(0.37)	1.23(0.49)	1.29(0.54)	1.62(0.9)	55.62 ***
	95%CI	1.22–1.27	1.12–1.16	1.18–1.27	1.24–1.34	1.51–1.74	
Passenger of a drugged driver (1–5)	Mean(SD)	1.22(0.6)	1.1(0.38)	1.19(0.49)	1.28(0.65)	1.66(1.05)	62.58 ***
	95%CI	1.19–1.24	1.07–1.12	1.14–1.23	1.22–1.34	1.52–1.79	

* *p* < 0.05; ** *p* < 0.01; *** *p* < 0.001.

**Table 4:** Adjusted logistic regression models for the risk of road traffic accidents (RTA) and severe RTA.

	Model 1 ° Outcome: ≥ 1 RTA(s)	Model 2 ° Outcome: ≥1 Severe RTA(s) °
	*Odds Ratio (95%CI)*	*Odds Ratio (95%CI)*
*Safe drivers (ref.)*	1	1
Average drivers	1.22 (0.93–1.60)	0.94 (0.46–1.92)
Careless drivers	1.78 * (1.35–2.35)	1.26 (0.64–2.45)
Reckless drivers	3.24 * (2.29–4.58)	2.15 * (1.06–4.40)

° Model adjusted by sex, age, average frequency of drive, and type of motor vehicle driven; * *p <* 0.001.

## Data Availability

The dataset generated and analyzed during the current study is available from the corresponding author on reasonable request.

## Supplementary Materials

The following are available online at https://www.mdpi.com/article/10.3390/ijerph18126362/s1, Table S1. Characteristics of the study population and of the four-clusters solution.
