# Supplementary material for: Profiles of Risky Driving Behaviors in Adolescent Drivers: A Cluster Analysis of a Representative Sample from Tuscany Region (Italy)
Source: Int J Environ Res Public Health. 2021 Jun 11;18(12):6362. doi: 10.3390/ijerph18126362 (PMC8296188; doi:10.3390/ijerph18126362)
Supplement: Supplementary file 1 [file ijerph-18-06362-s001.zip › ijerph-1238360-supplementary.pdf]

Table S1. Characteristics of the study population and of the four clusters solution

Characteristics		Total		Safe Drivers		Average Drivers		Careless Drivers		Reckless & Impaired Drivers	
		N	***%***	***N***	***%***	***N***	***%***	***N***	***%***	***N***	***%***
Sex	*Male*	1,473	68.1	685	69.5	326	69.2	293	63.1	169	69.8
	Female	689	31.9	300	30.5	145	30.8	171	36.9	73	30.2
Age	*14*	185	8.6	145	14.7	23	4.9	16	3.4	1	0.4
	15	291	13.5	206	20.9	35	7.4	30	6.5	20	8.3
	16	389	18.0	236	24.0	76	16.1	56	12.1	21	8.7
	17	383	17.7	216	21.9	75	15.9	63	13.6	29	12.0
	18	914	42.3	182	18.5	262	55.6	299	64.4	171	70.7
Educational level of the mother	*Pre-school education*	27	1.3	15	1.7	4	0.9	6	1.4	2	0.9
	Primary education	29	1.4	8	0.9	5	1.1	9	2.0	7	3.0
	Lower secondary education	472	23.1	186	20.5	111	24.4	115	26.0	60	25.5
	Upper secondary education	1,012	49.6	475	52.3	227	50.0	197	44.5	113	48.1
	Bachelor’s degree	99	4.9	40	4.4	18	4.0	28	6.3	13	5.5
	Master’s degree or higher	401	19.7	184	20.3	89	19.6	88	19.9	40	17.0
Educational level of the father	*Pre-school education*	39	1.9	22	2.4	5	1.1	5	1.1	7	3.1
	Primary education	48	2.4	11	1.2	12	2.7	13	3.0	12	5.2
	Lower secondary education	728	36.1	299	33.1	176	38.9	154	35.4	99	43.2
	Upper secondary education	869	43.1	401	44.5	194	42.9	192	44.1	82	35.8
	Bachelor’s degree	62	3.1	33	3.7	9	2.0	12	2.8	8	3.5
	Master’s degree or higher	272	13.5	136	15.1	56	12.4	59	13.6	21	9.2
Occupational status of the mother	*Unemployed*	474	23.1	234	25.2	92	20.1	99	22.4	49	21.8
	Salaried/pensioner	1,579	76.9	696	74.8	365	79.9	342	77.6	176	78.2
Occupational status of the father	*Unemployed*	68	3.4	31	3.4	11	2.5	15	3.5	11	5.2
	Salaried/pensioner	1,916	96.6	872	96.6	436	97.5	409	96.5	199	94.8
Parental family status	*Married/living together*	1,757	82.3	827	84.7	378	81.5	368	80.3	184	78.0
	Divorced/separated/widowed	377	17.7	149	15.3	86	18.5	90	19.7	52	22.0
Family relationship	*Very poor*	32	1.5	10	1.0	8	1.7	5	1.1	9	3.8
	Poor	44	2.0	18	1.8	6	1.3	14	3.1	6	2.5
	Fair	177	8.2	70	7.1	46	9.8	33	7.2	28	11.7
	Good	800	37.3	367	37.4	166	35.5	181	39.4	86	36.0
	Very good	1,094	51.0	516	52.6	242	51.7	226	49.2	110	46.0
Peer relationship	*Very poor*	14	0.7	8	0.8	3	0.6	1	0.2	2	0.8

	Poor	17	0.8	9	0.9	4	0.9	2	0.4	2	0.8
	Fair	154	7.2	72	7.3	28	6.0	30	6.5	24	10.0
	Good	992	46.1	453	46.2	231	49.4	211	45.7	97	40.4
	Very good	974	45.3	439	44.8	202	43.2	218	47.2	115	47.9
School performance	*Very poor*	42	2.0	17	1.7	8	1.7	10	2.2	7	2.9
	Poor	108	5.0	39	4.0	18	3.8	22	4.8	29	12.1
	Fair	584	27.1	251	25.6	121	25.9	131	28.4	81	33.8
	Good	1,167	54.2	553	56.3	258	55.1	248	53.7	108	45.0
	Very good	251	11.7	122	12.4	63	13.5	51	11.0	15	6.3
School year failure	*No*	1,667	77.4	840	85.5	365	77.8	334	72.6	128	53.1
	Yes	486	22.6	143	14.5	104	22.2	126	27.4	113	46.9
Nervousness in the last month	*Never*	98	4.5	56	5.7	18	3.8	11	2.4	13	5.4
	Rarely	432	20.0	236	24.0	90	19.2	71	15.3	35	14.5
	Sometimes	822	38.1	380	38.7	179	38.2	178	38.4	85	35.3
	Often	673	31.2	267	27.2	156	33.3	168	36.3	82	34.0
	Very often	130	6.0	43	4.4	26	5.5	35	7.6	26	10.8
Hopelessness in the last month	*Never*	1,048	48.8	510	52.1	232	49.5	204	44.3	102	42.3
	Rarely	489	22.8	226	23.1	106	22.6	100	21.7	57	23.7
	Sometimes	355	16.5	140	14.3	77	16.4	100	21.7	38	15.8
	Often	187	8.7	69	7.0	41	8.7	45	9.8	32	13.3
	Very often	70	3.3	34	3.5	13	2.8	11	2.4	12	5.0
Restlessness in the last month	*Never*	306	14.2	177	18.1	59	12.6	39	8.4	31	12.8
	Rarely	601	28.0	291	29.7	119	25.5	130	28.1	61	25.2
	Sometimes	712	33.1	313	32.0	169	36.2	149	32.3	81	33.5
	Often	440	20.5	159	16.2	111	23.8	118	25.5	52	21.5
	Very often	91	4.2	39	4.0	9	1.9	26	5.6	17	7.0
Sadness in the last month	*Never*	1,417	65.9	674	68.7	310	66.2	292	63.5	141	58.5
	Rarely	375	17.4	146	14.9	90	19.2	88	19.1	51	21.2
	Sometimes	224	10.4	100	10.2	40	8.5	54	11.7	30	12.4
	Often	105	4.9	48	4.9	24	5.1	22	4.8	11	4.6
	Very often	29	1.3	13	1.3	4	0.9	4	0.9	8	3.3
Exhaustion in the last month	*Never*	729	34.1	364	37.2	164	35.2	131	29.0	70	28.9
	Rarely	648	30.3	302	30.8	135	29.0	142	31.4	69	28.5
	Sometimes	440	20.6	178	18.2	110	23.6	102	22.6	50	20.7
	Often	239	11.2	102	10.4	43	9.2	59	13.1	35	14.5
	Very often	83	3.9	33	3.4	14	3.0	18	4.0	18	7.4

Worthlessness in the last month	*Never*	1,389	65.1	640	65.8	308	66.2	291	63.8	150	62.5
	Rarely	333	15.6	158	16.3	66	14.2	72	15.8	37	15.4
	Sometimes	229	10.7	91	9.4	49	10.5	63	13.8	26	10.8
	Often	117	5.5	57	5.9	29	6.2	23	5.0	8	3.3
	Very often	65	3.0	26	2.7	13	2.8	7	1.5	19	7.9
Sleep quality	*Deep/restful*	939	44.5	454	47.3	202	43.6	180	39.6	103	43.8
	Light	826	39.1	379	39.5	186	40.2	190	41.8	71	30.2
	Interrupted	347	16.4	126	13.1	75	16.2	85	18.7	61	26.0
Pathological gambling	*No*	1,903	89.3	892	92.0	429	92.3	402	87.8	180	75.6
	Yes	228	10.7	78	8.0	36	7.7	56	12.2	58	24.4
Smoking habit	*Regular smoker*	530	24.6	147	15.0	100	21.4	148	31.9	135	56.7
	Occasional/social smoker	315	14.6	122	12.4	72	15.4	84	18.1	37	15.5
	Non-smoker	1,306	60.7	712	72.6	296	63.2	232	50.0	66	27.7
Being drunk in the last year	*No*	852	41.2	509	54.6	171	38.1	137	30.4	35	15.0
	Yes	1,214	58.8	423	45.4	278	61.9	314	69.6	199	85.0
Binge drinking in the last month	*No*	1,204	58.0	648	68.7	264	58.7	228	50.4	64	27.7
	Yes	872	42.0	295	31.3	186	41.3	224	49.6	167	72.3
Drug use in the last month	*No*	1,621	75.6	820	83.8	354	75.2	327	71.1	120	50.8
	Yes	524	24.4	158	16.2	117	24.8	133	28.9	116	49.2
Bullying behavior in the last year	*No*	1,869	87.5	866	89.4	425	90.6	396	86.3	182	76.2
	Yes	267	12.5	103	10.6	44	9.4	63	13.7	57	23.8
Physical activity	*Less than 1 time a week*	189	9.0	77	8.0	46	10.0	37	8.2	29	12.6
	1 or more times a week	1,908	91.0	882	92.0	412	90.0	413	91.8	201	87.4
Age of the first sexual intercourse	*No sexual initiation*	1,021	48.3	627	65.3	198	43.2	162	35.4	34	14.2
	After age 13	827	39.1	246	25.6	212	46.3	236	51.6	133	55.6
	Before age 13	266	12.6	87	9.1	48	10.5	59	12.9	72	30.1
Use of alcohol or drug before the last sexual intercourse	*No*	889	81.3	289	85.8	229	87.1	244	83.0	127	63.5
	Yes	205	18.7	48	14.2	34	12.9	50	17.0	73	36.5
Use of condom during the last sexual intercourse	*No*	434	38.9	105	30.2	85	32.1	133	44.6	111	54.1
	Yes	682	61.1	243	69.8	180	67.9	165	55.4	94	45.9
Average frequency of driving	*6 day a week/every day*	1,386	64.1	499	50.7	325	69.0	346	74.6	216	89.3
	2-5 days a week	587	27.2	349	35.4	120	25.5	94	20.3	24	9.9
	1 day a week	189	8.7	137	13.9	26	5.5	24	5.2	2	0.8
Passenger of a	*Never*	1,680	79.4	840	86.8	366	79.9	340	74.9	134	57.0

drunk driver	*Once a month*	373	17.6	120	12.4	80	17.5	101	22.2	72	30.6
	A few times a month	50	2.4	8	0.8	11	2.4	11	2.4	20	8.5
	A few times a week	4	0.2	0	0.0	1	0.2	1	0.2	2	0.9
	Several times a week	8	0.4	0	0.0	0	0.0	1	0.2	7	3.0
Passenger of a drugged driver	*Never*	1,802	84.6	899	92.5	393	85.2	363	79.3	147	61.8
	Once a month	235	11.0	58	6.0	50	10.8	73	15.9	54	22.7
	A few times a month	57	2.7	9	0.9	17	3.7	12	2.6	19	8.0
	A few times a week	22	1.0	6	0.6	1	0.2	7	1.5	8	3.4
	Several times a week	13	0.6	0	0.0	0	0.0	3	0.7	10	4.2

Table S2. Logistic regression models for the risk of road traffic accidents (RTA) and severe RTA

	**Model 1**<br>*Outcome: ≥ 1 RTA(s)*	**Model 2**<br>*Outcome: ≥1 severe RTA(s)*
	Odds ratio (95%CI)	*Odds ratio (95%CI)*
Clusters		
Safe drivers (ref.)	1	1
Average drivers	1.22<br>(0.93-1.60)	0.94<br>(0.46-1.92)
Careless drivers	1,78**<br>(1.35-2.35)	1.26<br>(0.64-2.45)
Reckless drivers	3.24**<br>(2.29-4.58)	2.15**<br>(1.06-4.40)
Sex		
Male	1	1
Female	0.58**<br>(0.47- 0.73)	0.96<br>(0.54 – 1.69)
Age		
≤14	1	1
15	2.9 **<br>(1.69 – 4.98)	0.71<br>(0.13-3.9)
16	4.13 **	1.39

	(2.45 – 6.94)	(0.29 -6.71)
17	5.75**<br>(3.42 – 9.68)	1.18<br>(0.24 – 5.66)
≥ 18	6.4 **<br>(3.73 – 10.98)	1.01<br>(0.2 – 5.05)
Average driving frequency		
6 day a week/every day	1	1
Several times a week	0.62 **<br>(0.49- 0.78)	0.80<br>(0.41-1.53)
Once a week	0.39 **<br>(0.25 – 0.60)	1.27<br>(0.41 – 0.39)
Type of motor vehicle drived		
Passenger car	1	1
Moped ≤ 50 cc	3.68 **<br>(2.65 – 5.12)	0.74<br>(0.34-1.59)
Motorbike 50 cc - 125 cc	3.63**<br>(2.45 – 5.36)	1.31<br>(0.55 – 3.10)
Motorbike ≥ 125 cc	3.13 **<br>(2.02-4.86)	1.13<br>(0.43-3.00)
Moped car ≤ 50 cc	2.09 *<br>(1.15- 3.81)	1.74<br>(0.5352 -5.67)

* *p< 0,05* ** *p< 0,001*
